# Dual-Modal Illumination System for Defect Detection of Aircraft Glass Canopies

**DOI:** 10.3390/s24206717

**Published:** 2024-10-18

**Authors:** Zijian Li, Yong Yao, Runyuan Wen, Qiyang Liu

**Affiliations:** School of Computer Science and Technology, Xidian University, Xi’an 710126, China; zjian.li@stu.xidian.edu.cn (Z.L.); wenrunyuan@stu.xidian.edu.cn (R.W.); 22031212452@stu.xidian.edu.cn (Q.L.)

**Keywords:** aircraft glass canopy, defect detection, image fusion, technical diagnostics

## Abstract

Defect detection in transparent materials typically relies on specific lighting conditions. However, through our work on defect detection for aircraft glass canopies, we found that using a single lighting condition often led to missed or false detections. This limitation arises from the optical properties of transparent materials, where certain defects only become sufficiently visible under specific lighting angles. To address this issue, we developed a dual-modal illumination system that integrates both forward and backward lighting to capture defect images. Additionally, we introduced the first dual-modal dataset for defect detection in aircraft glass canopies. Furthermore, we proposed an attention-based dual-branch modal fusion network (ADMF-Net) to enhance the detection process. Experimental results show that our system and model significantly improve the detection performance, with the dual-modal approach increasing the mAP by 5.6% over the single-modal baseline, achieving a mAP of 98.4%. Our research also provides valuable insights for defect detection in other transparent materials.

## 1. Introduction

Transparent materials are widely used in canopies, windshields, and windows of aircraft. As external structural components of the aircraft and vision windows for pilots, these materials must possess excellent mechanical strength and optical properties to ensure operational safety. However, due to the complexities inherent in the manufacturing processes and the continuous exposure to environmental factors, such as high speed dust impact and abrasion during flight [1], surface defects, such as cracks, scratches and pits, inevitably develop over time. These defects can interfere with the pilot’s field of vision and, in more severe cases, present significant structural safety risks [2,3].

Currently, the inspection and maintenance of aircraft transparent materials rely heavily on manual visual inspections. This approach is not only labor-intensive and time-consuming but also prone to human error, resulting in inconsistent outcomes and the potential for missed defects [4]. Therefore, the development of an automated, accurate and efficient defect detection system is crucial for improving the quality control and maintenance processes for aircraft transparent materials.

In recent years, machine vision technology has been increasingly applied to defect detection and quality control in industrial manufacturing. This paper seeks to bridge the gap between research and industrial application by introducing a novel defect detection system for aircraft glass canopies, along with a defect dataset and detection model.

Our contributions can be summarized as follows:

1. An automated prototype system with a dual-modal illumination structure is proposed. For the same sampling point, the system sequentially uses forward lighting and backward lighting to capture defect images. By integrating the information from both types of images, more accurate defect detection is achieved.
2. A curated dataset is presented, which, to the best of our knowledge, is the first benchmark in the field of defect detection for aircraft transparent materials.
3. A dual-modal baseline is designed and proved to be competitive, in which we designed two fusion detection methods: a data-level fusion method named RGB Channel Fusion, and an attention-based dual-branch modal fusion network using feature-level fusion. Both methods achieved excellent results on our dataset.

## 2. Related Work

Product defect detection is a crucial aspect of quality control in the manufacturing industry. Automated Optical Inspection (AOI), as a non-contact testing technique, is widely used across various product production lines [5]. However, research on defect detection systems for aircraft transparent materials lags far behind. Defect detection techniques in other fields can serve as valuable references for this study.

### 2.1. Traditional Machine Vision Method for Defect Detection in Transparent Materials

Traditional defect detection for transparent materials often relies on specialized optical or acoustic instruments [6,7]. With the development of image processing technology and machine learning, machine vision methods have started to be applied. These methods use conventional image processing techniques, such as edge detection, texture analysis, or shape matching, to extract and identify defect objects.

For instance, Zhou X et al. [8] proposed a defect detection strategy for glass bottle bottoms based on wavelet transform and multi-scale filtering algorithms, which effectively reduces the impact of texture on defect detection. Jian C et al. [9] captured images of mobile phone cover glass under coaxial parallel lighting and used template subtraction and projection methods for defect detection.

Traditional machine learning-based defect detection methods often rely on the comparison between defect images and defect-free templates [10], making them very sensitive to noise, background interference, and uneven lighting. These methods are mainly suitable for defect detection in controlled scenarios.

### 2.2. Deep Learning-Based Visual Defect Detection

In recent years, deep learning-based machine vision methods have been widely applied in the field of defect detection. Compared to traditional machine learning methods, deep learning offers more complex model fitting capabilities, allowing it to learn high-level abstract features [11]. This results in better accuracy and generalization in defect detection tasks.

For instance, Park J et al. [12] proposed a defect detection system for curved glass of mobile phones based on weighted multi-neural networks, effectively utilizing multi-channel measurement data. Cheng L et al. [13] introduced an infrared thermography-based generative adversarial network (IRT-GAN) for non-destructive testing of carbon fiber and glass fiber polymer composites. Zhu Y et al. [14] introduced a ResNeSt network based on deformable convolution to detect fine features of surface defects on mobile phones, addressing various defect shapes. They also used RoI Align to reduce localization errors, particularly for scratch defects with high aspect ratios.

R-CNN algorithms, including Fast R-CNN and Faster R-CNN, are well-established two-stage object detection algorithms. Pan Z et al. [15] developed an automatic surface scratch inspection system using a rail-based microscopic camera scanning system for detecting defects in architectural glass curtain walls. They used a Mask R-CNN network model to achieve pixel-level instance segmentation.

The YOLO series algorithms, as the most typical representatives of one-stage object detection algorithms, transform the object detection process into a regression task, significantly speeding up detection and maintaining a leading position in real-time object detection tasks [16]. Zhang M et al. [17] adopted an improved YOLOv5 algorithm for defect detection in solar cells, incorporating deformable convolutions, ECA-Net attention mechanism, Mosaic and MixUp data augmentation, and K-means++ for anchor box clustering, improving the algorithm’s mAP by 7.85% and achieving a detection speed of 36.24 FPS, meeting real-time detection requirements. Xie Y et al. [18] proposed a feature-enhanced surface defect detection algorithm (FE-YOLO) for practical industrial applications, combining depthwise separable convolution with dense connections and introducing an improved feature pyramid network to enhance spatial position correlation in multi-scale detection layers, achieving high accuracy while reducing model weight.

### 2.3. Multi-Modal Fusion in Object Detection

Multi-modal information fusion has gained attention in the field of object detection. These methods leverage the complementary information from different modalities to construct more comprehensive and enriched features, thereby improving detection accuracy.

In multi-modal image object detection, fusion methods are generally categorized into three types [19]: data-level fusion (early fusion), feature-level fusion (intermediate fusion), and decision-level fusion (late fusion).

A typical example is the fusion of visible light and infrared images for nighttime pedestrian detection. Data-level fusion involves combining visible light and infrared images into a single image using specific algorithms or multiple data channels before feeding them into the detection model. Decision-level fusion uses two separate models to detect visible and infrared images independently, focusing on post-processing the outputs of the two models. Feature-level fusion uses parallel branches to extract independent features from images at different scales, embedding a feature fusion layer between the feature layers, and using the fused features for detection.

Compared to decision-level fusion, a well-designed feature-level fusion method enables the model to learn the correlations and differences between different modalities, and reduces redundant computations.

Multi-modal fusion object detection has been applied and validated in various fields. Examples include RGB and infrared thermal image fusion for object detection [20,21], RGB and radar information fusion for assisted driving [22], polarization and infrared imaging fusion for PCB defect detection [23], and multi-spectral image fusion for medical image segmentation [24].

## 3. Methodology

### 3.1. Dual-Modal Illumination Structure

**Figure 1:**
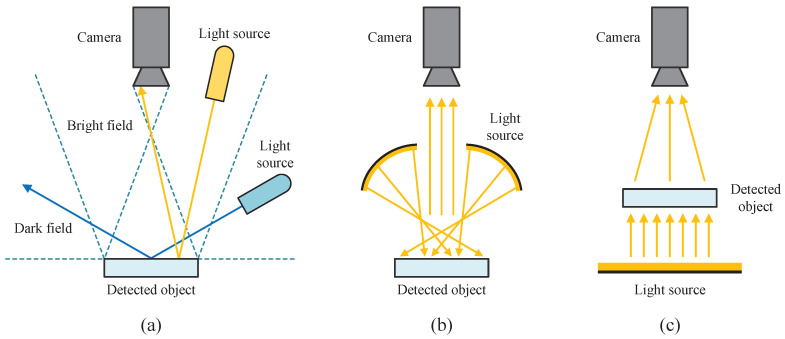
Schematic diagram of the light path of common illumination structures. (**a**) Bright field and dark field forward lighting. (**b**) Scattering forward lighting. (**c**) Backward lighting.

In industrial machine vision defect detection, an appropriate illumination structure design is crucial for capturing defect images. Among various illumination structures, forward lighting is the most widely used. Depending on whether the light is reflected directly into the camera lens, which is usually controlled by the incident angle of the light source, forward lighting can be divided into bright field forward lighting and dark field forward lighting, as shown in Figure 1a. Additionally, for highly reflective surfaces, scattering forward lighting can effectively reduce the strong reflections [25]. This type of lighting is usually achieved using a dome structure or multi-angle light source to provide uniform illumination, as shown in Figure 1b.

In contrast to forward lighting, the backward lighting method places the light source behind the object, as depicted in Figure 1c. Backward lighting is commonly used for defect detection in transparent materials as it can highlight internal defects, but it is less sensitive to surface defects [26].

**Figure 2:**
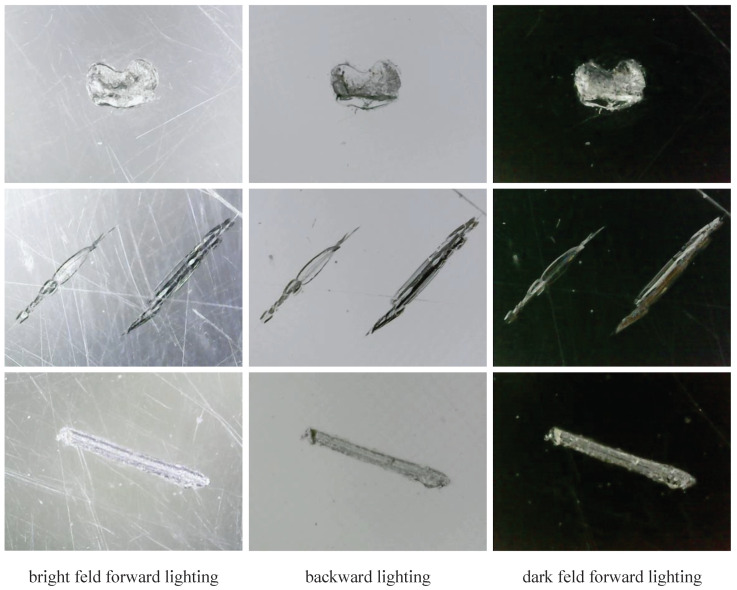
Typical defect samples under different illumination structures.

To design an appropriate illumination structure for this study, we captured images of typical defects under different lighting conditions, with some of the results shown in Figure 2.

Through the analysis of these images collected, we found that bright field forward lighting easily captures surface scratch-type defects but is susceptible to reflections and uneven lighting. Backward lighting effectively reveals the depth information of defects, aiding in the analysis of defect severity and classification. However, it tends to lose surface detail and some boundary contour information. The effect of dark field forward lighting is similar to backward lighting, resembling an inverted operation.

We realized that the information provided by multiple modalities is complementary, contributing to a more accurate assessment of defect conditions. Our subsequent experiments in this paper also verified that the multi-modal defect detection method has significantly improved performance compared to the single-modal method.

### 3.2. Image Acquisition Platform

Considering the practical feasibility in industrial inspection environments, we ultimately chose to use a dual-modal illumination structure, employing both forward lighting and backward lighting to capture and detect defects. For the implementation of forward lighting, we use a ring light source to achieve more uniform illumination and reduce reflections. For backward lighting, we use a planar light source.

**Figure 3:**
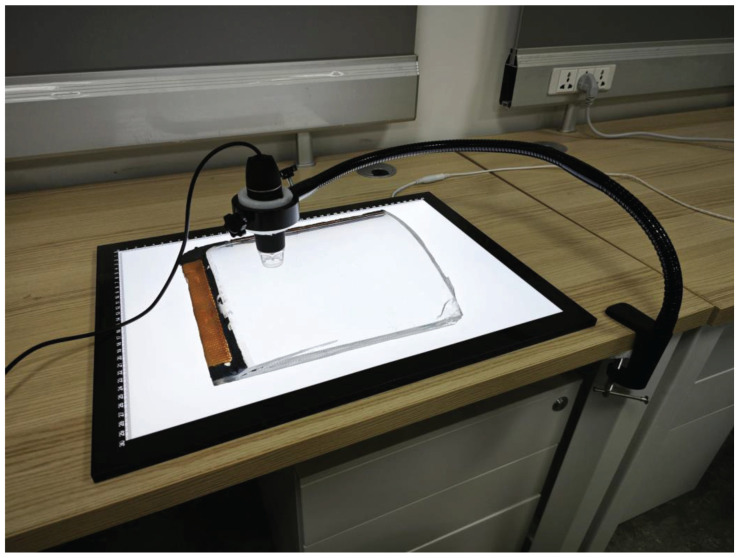
Prototype dual-modal illumination image acquisition platform.

We built a prototype dual-modal illumination image acquisition platform, as shown in Figure 3. This platform mainly consists of a digital microscope, a flexible arm holder, a backlight panel, and a ring light source.

The defects on glass canopies mostly range in diameter from 80 μm to 500 μm, making it impractical to use conventional CCD cameras for imaging. Therefore, we use a digital microscope as the image acquisition device. This device features an adjustable focal length ranging from 8 mm to 60 mm, with a magnification range of 50× to 1000×, and a photo resolution of 1280×720. During image acquisition, the focal length was set to 20 mm, with an approximate magnification of 400×.

Due to the necessity of maintaining a fixed position between the camera lens and the sample when capturing images under two lighting conditions, we used a flexible arm holder as the support device. This device has a length of 60 cm and a maximum load capacity of 1.0 kg, providing sufficient stability for the image acquisition process.

**Figure 4:**
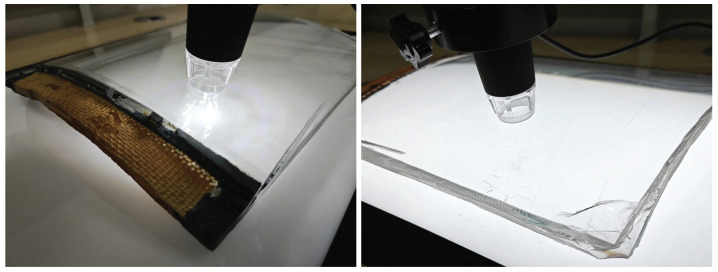
Forward lighting and backward lighting implementation.

The forward lighting method uses 8 ring-shaped LED lights positioned around the camera lens. The backward lighting method uses a 30 cm × 42 cm backlight panel with a brightness of 500 nits and a color temperature of 6500 K. The two light sources are enabled alternately by an electrically controlled switch during image acquisition. See Figure 4.

### 3.3. Dataset Construction

**Figure 5:**
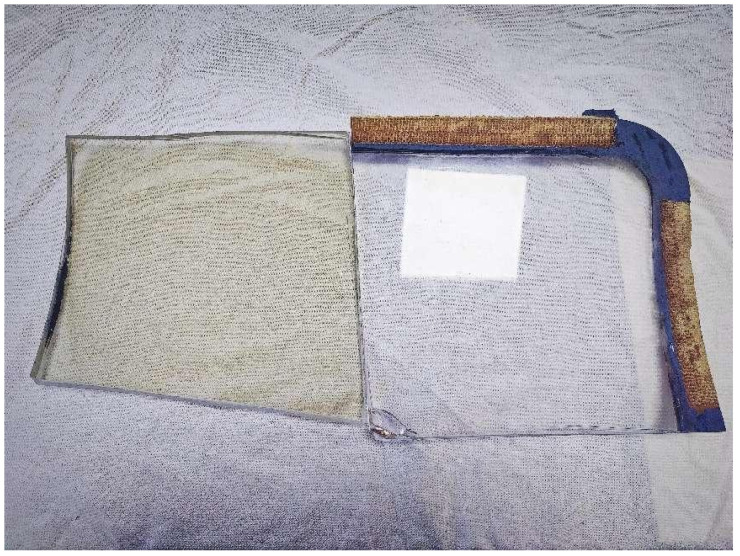
Aircraft glass canopy samples.

Due to the specialized nature of the research, publicly available defect sample data for aircraft glass canopies are not currently accessible. Therefore, we collected data from our own samples of real aircraft cockpit canopies, which served as the source for our subsequent experiments. See Figure 5.

**Figure 6:**
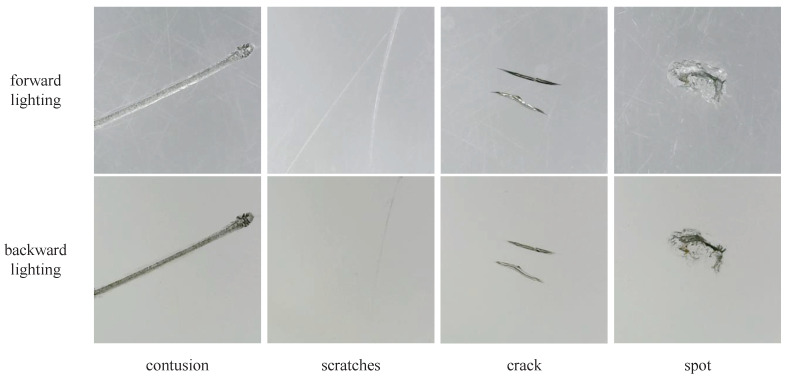
Typical images of four types of defects.

Based on the real aircraft glass canopies samples, we collected a total of 438 raw images, covering 219 defect points using two different lighting methods. Through image analysis and relevant experience, we categorized the collected defects into four types: contusion, scratches, crack, and spot. These categories essentially cover all possible defect types that may appear on the aircraft glass canopy. Typical images are shown in Figure 6.

Considering that many of the detected target objects are slender, that is, they have high aspect ratios, large image spans, and may be interlaced, we choose to use oriented bounding boxes (OBB) for annotation and detection.

We use X-AnyLabeling [27] (version 2.3.5) for data annotation, which allows the use of existing segmentation models to improve annotation efficiency. We choose the Segment Anything (ViT-Large) model for pre-labeling, which provides preparatory polygon boundaries of the selected defects. Then, a script is written to calculate the minimum enclosing rectangle for each defect, resulting in a more precise rotated bounding box.

Manual adjustments are also necessary. This is because some defects are only clearly outlined under a specific lighting condition. For example, scratch defects are usually easier to observe under forward lighting. However, some small spot-type defects that are not apparent under forward lighting are very prominent under backward lighting. Additionally, there are also subtle differences in the contour boundaries of the defects under the two types of lighting, and in that case, the union outer contour will be selected. We check each annotation file to ensure the accuracy and usability of the dataset.

To address the limited number of defect images available for aviation transparent parts, we also adopted several data augmentation methods. In order to improve the defect detection performance under any rotation angle, we performed pre-rotation augmentation on the defect samples. In this process, rotation and mirroring operations are simultaneously applied to the images and their labels to avoid redundant annotation work. By adjusting the step size of the rotation angle, the augmentation rate can be controlled. For example, using 90-degree rotations and mirroring operations, an 8× augmentation can be achieved without information loss. For general angles, the background color RGB(114, 114, 0) is filled in the blank areas to reduce interference. Moreover, since the standard OBB format does not allow point coordinates to be located outside the image boundaries, labels that overflow during the rotation process need to be adjusted or deleted. The correction method for overflow labels is as follows:

1. If all four points of the bounding box are outside the field of view, delete the target (process ends), otherwise go to step 2.
2. If all four points of the bounding box are within the field of view, no adjustment is required (process ends), otherwise go to step 3.
3. Select a point that is outside the field of view. Calculate the distance between the point and its adjacent two points to find the long and short sides connected to the point on the rectangle. Translate the short side along the direction of the long side until the point falls on the image boundary. The translation amount is determined by solving a linear programming problem. After adjustment, return to step 2 to re-evaluate.

By following these steps, we ensure that the labels are correctly normalized. Additionally, during the training process, we employed the Mosaic-4 data augmentation method, which includes random image combinations, random scaling and translation, contrast adjustments, exposure adjustments, and noise addition.

The final dataset of aircraft glass canopy defects, named ag_dual_obb, includes four types of defects, totaling 1752 defect points and 4784 defect objects. Each defect point includes two images: a forward lighting image and a backward lighting image, which are strictly spatially aligned. The dataset is divided into a training set with 1576 image pairs and a test set with 176 image pairs, following a 9:1 split ratio. See Table 1.

We also created some variant datasets for extra experiments, which follow similar naming. For example, the dataset labeled with standard rectangular box is named ag_dual_rect, and the dataset obtained through RGB Channel Fusion is named ag_composite_obb.

### 3.4. RGB Channel Fusion Method

**Figure 7:**
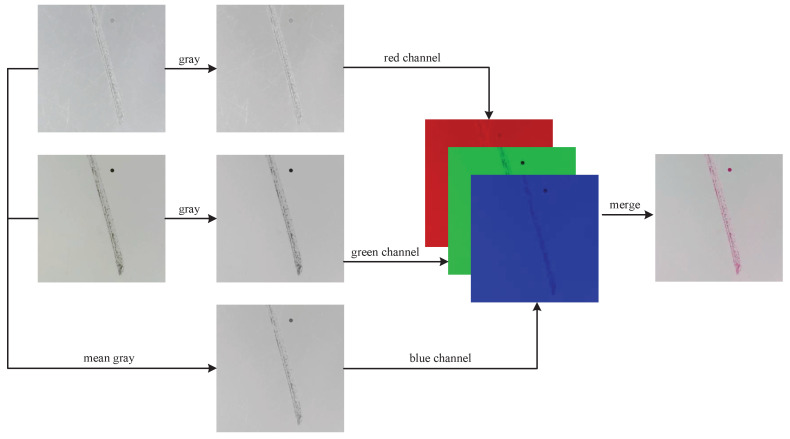
RGB Channel Fusion method.

To initially validate the effectiveness of fusion detection for this task, we first designed a data-level fusion method named RGB Channel Fusion, as shown in Figure 7. In the RGB Channel Fusion method, the forward lighting image and the backward lighting image are each converted to grayscale. These grayscale images are then assigned to the red channel and green channel of the fused image, respectively, while the blue channel is obtained by averaging the two grayscale images.

The design rationale for the RGB Channel Fusion method is based on the fact that, for this specific task, grayscale conversion hardly diminishes the image information. Moreover, the averaging of images can serve as a simple yet effective image fusion technique. In addition, the resulting color of the fused image can provide a reference for our data annotation work. For example, a dark red color usually means deeper damage, which helps distinguish surface scratches from more serious contusions.

### 3.5. Attention-Based Dual-Branch Modal Fusion Network (ADMF-Net)

**Figure 8:**
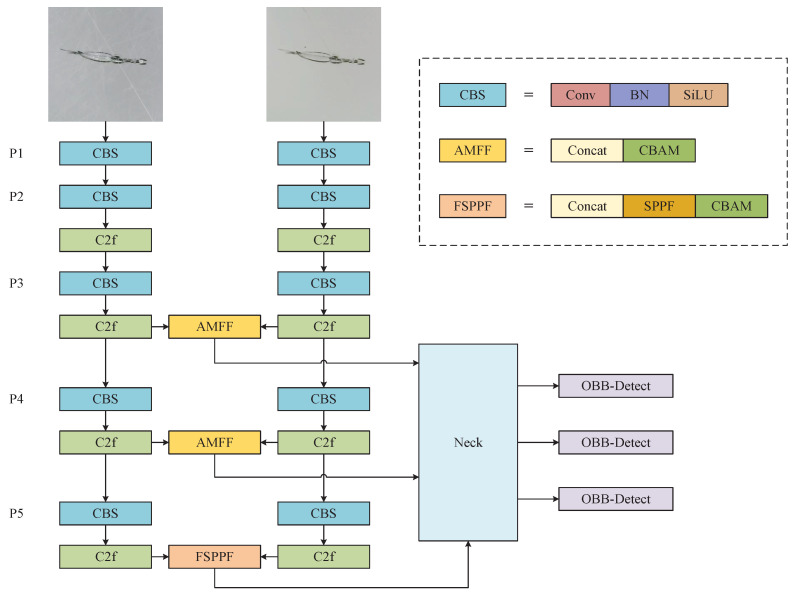
The architecture of the proposed ADMF-Net.

To fully utilize the multimodal information obtained under different lighting conditions, this paper proposes an attention-based dual-branch modal fusion network (ADMF-Net) for defect detection. The overall architecture of the network is shown in Figure 8. It consists of two independent feature extraction backbones, feature fusion modules (AMFF, Attention-based Multi-modal Feature Fusion Module), a multi-scale feature pyramid pooling module (FSPPF, Fusion-based Spatial Pyramid Pooling - Fast), and detection layers.

In this network, we use two CSPDarknet-like feature extraction backbones, as proposed in YOLOv8, to extract multi-scale features from the forward lighting and backward lighting modalities separately. Next, two AMFF modules are inserted between the two backbones, applied to the P3 and P4 layers, to obtain fused features with different receptive fields. Then, the P5 layers from both modalities are connected to a spatial pyramid pooling layer, FSPPF. Finally, the three scales of features are fed into the detection layers, using an OBB detection head to predict the defects with rotated bounding boxes.

The AMFF module consists of a Concat module and a convolutional block attention module (CBAM). The feature fusion process involves three steps.

First, the Concat operation is applied to the feature maps of the two modalities to create the initial fused feature.

(1) $$F=\left([F^{\mathrm{forward}};F^{\mathrm{backward}}]\right)$$

Second, a channel attention module (CAM) is used to adjust the channel weights between and within the two modalities. The input feature map $F\in R^{\left(C\times H\times W\right)}$ is transformed into C×1×1 using maximum pooling and average pooling, respectively. These two feature vectors are then fed into a shared MLP for learning the attention weights of channels. The results are combined using the addition operation and normalized by the sigmoid function. Multiplying the final weight coefficient $M_{c}\in R^{\left(C\times 1\times 1\right)}$ with the original feature map gives the new feature map $F^{\prime }$.

(2) $$\begin{array}{cc} M_{c}\left(F\right) & =\sigma (\mathrm{MLP}(\mathrm{AvgPool}(F))+\mathrm{MLP}(\mathrm{MaxPool}(F)))\\ \; & =\sigma \left(W_{1}\left(W_{0}\left(F_{\mathrm{avg}}^{c}\right)\right)+W_{1}\left(W_{0}\left(F_{\max}^{c}\right)\right)\right) \end{array}$$

(3) $$F^{\prime }=M_{c}\left(F\right)⊗F$$

Third, the fused feature is fed into the spatial attention module (SAM) to enhance the attention on the important defect areas and to reduce the weight of background areas. In this process, maximum and average pooling are applied to each channel to obtain two feature maps of 1×H×W, then concatenated by channel and sent into a 7×7 convolution layer. After use of the sigmoid activation function, the spatial attention map $M_{s}\in R^{\left(1\times H\times W\right)}$ is obtained. Lastly, the $M_{s}$ and $F^{\prime }$ are multiplied to obtain the final fused feature map $F^{\prime \prime }$.

(4) $$\begin{array}{cc} M_{s}\left(F\right) & =\sigma \left(f^{7\times 7}\left(\left[\mathrm{AvgPool}\left(F\right);\mathrm{MaxPool}\left(F\right)\right]\right)\right)\\ \; & =\sigma \left(f^{7\times 7}\left([F_{\mathrm{avg}}^{s};F_{\max}^{s}]\right)\right) \end{array}$$

(5) $$F^{\prime \prime }=M_{s}\left(F^{\prime }\right)⊗F^{\prime }$$

The FSPPF module first concatenates the outputs of the P5 layers from the two feature extraction backbones, and then passes it into the SPPF (Spatial Pyramid Pooling-Fast) module. Then, similarly, the attention mechanism is applied to refine and produce the final feature map. The output of FSPPF, along with the outputs from the P3 and P5 layers’ AMFF modules, serves as the input to the neck layer.

## 4. Experiments

### 4.1. Evaluation Metrics

We evaluate the model’s detection performance using the mean average precision (mAP). Precision is the ratio of true positives (TP) to the sum of true positives and false positives (FP), indicating the accuracy of positive predictions. Recall is the ratio of true positives to the sum of true positives and false negatives (FN), reflecting how well the model detects all relevant instances. Average precision (AP) measures the area under the precision-recall curve, denoted as p(r), which describes the relationship between precision and recall as the confidence threshold changes. The final mAP is obtained by averaging the AP values across all classes. In this study, we use both the conventional mAP50, which evaluates at an intersection over union (IoU) threshold of 0.5, and the stricter mAP50-95, which averages over multiple IoU thresholds from 0.5 to 0.95 in steps of 0.05.

(6) $$\begin{array}{c} \mathrm{Precision}=\frac{TP}{TP+FP}\\ \mathrm{Recall}=\frac{TP}{TP+FN}\\ AP=\int_{0}^{1}p\left(r\right)dr\\ mAP=\frac{1}{N}\sum AP_{i} \end{array}$$

### 4.2. Implementation Details

The training environment for the experiments was configured with Python version 3.10.14 and PyTorch version 2.2.2, with CUDA version 11.8 for GPU acceleration. The Ultralytics version 8.2.2 was employed to support model implementation. The hardware setup included an AMD Ryzen™ 7 7800X3D CPU with 8 cores and 16 threads, 48GB of memory, and a NVIDIA® GeForce RTX™ 4080 GPU with 16GB of VRAM. During the training phase, we adopted the hyperparameter settings shown in Table 2.

### 4.3. Results and Discussion

#### 4.3.1. Baseline and Dual-Modal Validity Discussion

First, we used YOLOv8m as the baseline model, training on the single-modal datasets to test the baseline performance. The results show that using only forward lighting images achieved a mAP50 of 92.8%, while using only backward lighting images achieved a mAP50 of 79% (see Table 3). However, as seen in the detailed results for each defect type, backward lighting significantly outperforms forward lighting in detecting contusions and spot defects. In contrast, its AP for scratches is only 23%, which considerably lowers its overall mAP (see Table 4).

Next, we trained the model on the ag_composite_obb dataset, which is created using the RGB Channel Fusion method. The results show that our simple channel fusion method achieved impressive improvements. The overall mAP reached 97.9%, which is close to the natural error margin of manual annotations. Furthermore, the AP for each defect type surpassed the highest levels achieved using a single lighting method, with only a slight decrease in AP50-95 for the crack defect category.

Although the overall performance of forward lighting is barely satisfactory, it cannot fully replace the detection capabilities of backward lighting. Combining both methods for defect detection is obviously more effective and holds potential for further improvement.

#### 4.3.2. ADMF-Net Experiment

**Figure 9:**
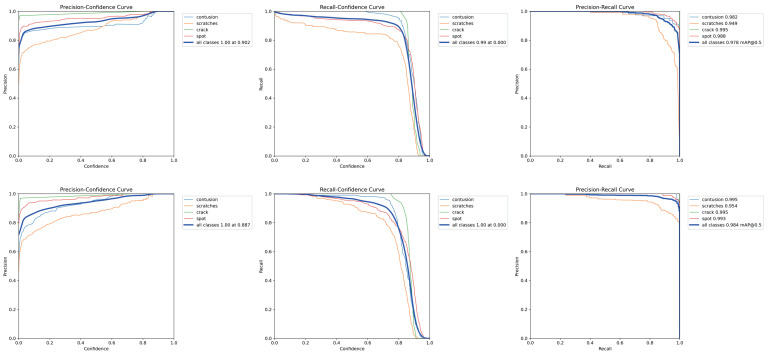
A comparison of the two proposed methods. First row: RGB Channel Fusion. Second row: ADMF-Net.

Our fusion model achieved excellent results on the ag_dual_obb dataset. The detection rates for the contusion, crack, and spot categories all exceeded 99%. The detection rate for scratches was 95.4%, which is consistent with the results of the RGB Channel Fusion method. Overall, compared to the RGB Channel Fusion method, the total mAP50 improved from 97.9% to 98.4%. As shown in Figure 9, our fusion model demonstrated a better precision-confidence curve and precision-recall curve, although there was a slight decrease in mAP50-95.

#### 4.3.3. Interpretability Experiment

**Figure 10:**
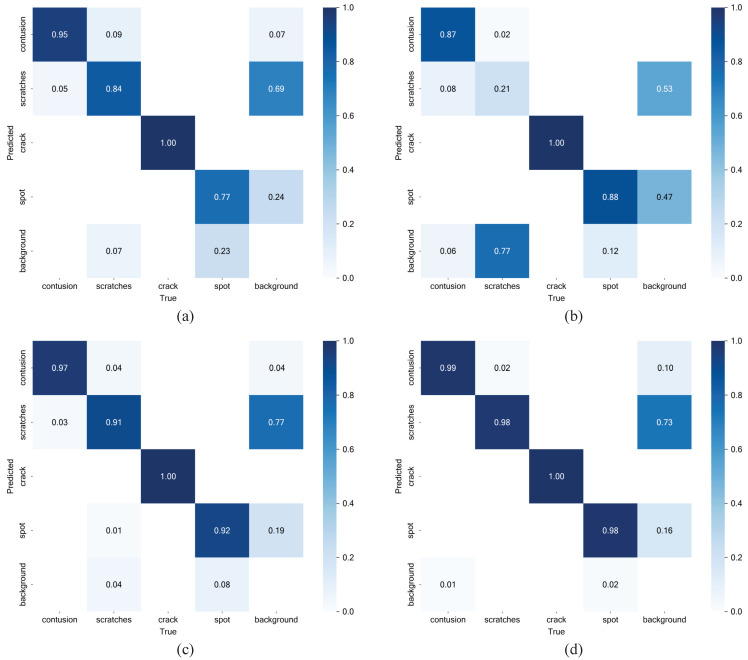
Normalized confusion matrix of single lighting methods and our fusion methods. (**a**) Forward lighting. (**b**) Backward lighting. (**c**) RGB Channel Fusion. (**d**) ADMF-Net.

A confusion matrix is a table used to evaluate classification models by comparing predicted and actual values. As shown in Figure 10, the model trained with forward lighting tends to misclassify lighter scratch defects as contusions and struggles to detect spot defects. For backward lighting, a significant number of scratch defects are incorrectly classified as background, which is the primary factor limiting its overall performance. These issues were significantly improved with the adoption of the fusion detection method.

#### 4.3.4. Comparative Analysis of Results

We conducted several comparative experiments to verify the effectiveness of our proposed method. See Table 5. For the selection of the third channel in the RGB Channel Fusion method, we experimented with using the difference image of the two modalities instead of the mean value. This resulted in a slight decrease in mAP.

To validate the effectiveness of our fusion model, we conducted ablation experiments. The results showed that after removing the attention mechanism, mAP50 remained relatively stable, but mAP50-95 significantly dropped by 5.2%.

Additionally, we conducted further experiments to evaluate the impact of different attention mechanisms on detection performance, such as EMA attention and a state-of-the-art model based on transformer cross-attention. The experimental results indicate that these more complex attention mechanisms could have negative effects. Our proposed model proves to be more effective for defect detection in aircraft glass canopies.

## 5. Conclusions

To address the issue of surface defect detection on aircraft glass canopies, where relying on a single-modal lighting condition often leads to missed or false detections, we have proposed a dual-modal illumination system for transparent material defect detection. This system utilizes both forward lighting and backward lighting to capture defect images, facilitating a more comprehensive and accurate defect detection process.

In addition, based on real aircraft glass canopy samples, we have collected the first dataset for defect detection in aircraft glass canopies. This dataset includes 1752 defect points captured under both forward and backward lighting conditions, totaling 4784 defect samples. These samples are categorized into four classes and annotated using the OBB format.

Furthermore, we proposed a data-level fusion method named RGB Channel Fusion, and a feature-level fusion model, the attention-based dual-branch modal fusion network (ADMF-Net). The model uses two feature extraction backbones to independently extract multi-scale features from the forward and backward lighting modalities. Next, through the attention-based multi-modal feature fusion block (AMFF) and improved SPPF module, the network adaptively integrates the multi-scale features from the two modalities. Finally, the features are fed into the detection layers, where an OBB detection head is used to provide the rotated bounding boxes of defects.

Experiments demonstrate the effectiveness of our proposed system and dual-modal detect methods. Compared to the baseline model YOLOv8m, which individually detects forward lighting and backward lighting images, our ADMF-Net model improved the detection accuracy by 5.6% and 19.4%, reaching a mAP of 98.4%. The proposed RGB Channel Fusion method also achieved a strong mAP of 97.9%. The comparative experiments also confirmed the superiority of our model.

Our research effectively addresses the issue of defect detection in aircraft glass canopies. Moreover, it provides a new methodological support for defect detection in other transparent materials. Exploring specific multi-modal detection networks remains a worthwhile issue.

## Figures and Tables

**Table 1:** Defect quantity statistics.

Index	Defect Type Name	Count
0	contusion	1104
1	scratches	1728
2	crack	232
3	spot	1720

**Table 2:** Hyperparametric configuration.

Parameter Name	Parameter Value
Epoch	300
Batch size	16
Image size	640×640
Data augmentation	Mosaic-4
Optimizer	SGD
Learning rate	0.01
Momentum	0.937
Weight decay	0.0005

**Table 3:** Experimental results overview.

Index	Dataset Name	Model	mAP50	mAP50-95
1	forward_only	YOLOv8m	0.928	0.641
2	backward_only	YOLOv8m	0.790	0.538
3	ag_composite_obb	RGB Channel Fusion (ours) ^1^	0.979	**0.691**
4	ag_dual_obb	ADMF-Net (ours)	**0.984**	0.669

^1^ Data-level fusion, using YOLOv8m as the back end. Note: In all experiments, the model scale was set to m if optional. Bold values indicate the best performance for each metric.

**Table 4:** Detailed AP results for the four types of defect.

Index	Contusion	Scratches	Crack	Spot
1	0.908/0.649	0.900/0.517	0.995/**0.743**	0.908/0.652
2	0.951/0.679	0.233/0.118	0.995/0.685	0.980/0.669
3	0.980/**0.716**	**0.957**/**0.556**	0.995/0.737	0.984/0.754
4	**0.995**/0.686	0.954/0.520	0.995/0.714	**0.993**/**0.756**

Note: For each data item, the values from left to right represent AP50 and AP50-95. Bold values indicate the best performance for each metric.

**Table 5:** Additional experimental results.

Index	Dataset Name	Model	mAP50	mAP50-95
5	ag_composite_diff_obb	RGB Channel Fusion ^1^	0.977	0.684
6	ag_dual_obb	ADMF-Net ^2^	0.983	0.639
7	ag_dual_obb	ADMF-Net ^3^	0.976	0.668
8	ag_dual_rect	ICAFusion [28] ^4^	0.963	0.640

^1^ Using differential image as the third channel. ^2^ Without attention mechanism, using only Concat operation. ^3^ Using EMA attention [29]. ^4^ Using transformer cross-attention.

## Data Availability

The data that support the findings of this study are publicly available for research purposes at the following GitHub repository: https://github.com/core128/AGDD (accessed on 14 October 2024).
